# Comparison of Estimated LDL Cholesterol Equations with Direct Measurement in Patients with Angiographically Confirmed Coronary Artery Disease

**DOI:** 10.3390/jcdd9100342

**Published:** 2022-10-07

**Authors:** Boqun Shi, Hao-Yu Wang, Dong Yin, Chenggang Zhu, Lei Feng, Hongjian Wang, Lei Jia, Rui Fu, Chenxi Song, Zhou Zhou, Yahui Lin, Weihua Song, Ke-Fei Dou

**Affiliations:** 1 Cardiometabolic Medicine Center, Department of Cardiology, Fuwai Hospital, Chinese Academy of Medical Sciences & Peking Union Medical College/National Center for Cardiovascular Diseases, Beijing 100037, China; 2 Coronary Heart Disease Center, Department of Cardiology, Fuwai Hospital, Chinese Academy of Medical Sciences & Peking Union Medical College/National Center for Cardiovascular Diseases, Beijing 100037, China; 3 State Key Laboratory of Cardiovascular Disease, Beijing 100037, China; 4 Center of Laboratory Medicine, Key Laboratory for Molecular Diagnostics of Cardiovascular Diseases, Fuwai Hospital, Chinese Academy of Medical Sciences & Peking Union Medical College/National Center for Cardiovascular Diseases, Beijing 100037, China

**Keywords:** LDL-C

## Abstract

**Background and aims:** Our goals in the study were to (1) quantify the discordance in LDL-C levels between equations (the Friedewald, Sampson, and Martin/Hopkins equations) and compare them with direct LDL-C (dLDL-C); and (2) explore the proportion of misclassified patients by calculated LDL-C using these three different equations. **Methods:** A total of 30,349 consecutive patients with angiographically confirmed coronary artery disease (CAD) were prospectively enrolled. Concordance was defined as if the LDL-C was <1.8 mmol/L with each pairwise comparison of LDL-C equations. Estimated LDL-C that fell into the same category as dLDL-C at the following levels: <1.4, 1.4 to 1.7, 1.8 to 2.5, 2.6 to 2.9, and ≥3.0 mmol/L was considered to have been correctly categorized. **Results:** The concordance was 96.3% (Sampson vs. Martin/Hopkins), 95.0% (Friedewald vs. Sampson), and 91.4% (Friedewald vs. Martin/Hopkins), respectively. This proportion fell to 82.4% in those with hypertriglyceridemia (TG ≥ 1.7 mmol/L). With an accurate classification rate of 73.6%, the Martin/Hopkins equation outperformed the Sampson equation (69.5%) and the Friedewald equation (59.3%) by a wide margin. **Conclusions:** Comparing it to the validated Martin/Hopkins equation, the Friedewald equation produced the lowest levels of LDL-C, followed by the Sampson equation. In the classification of LDL-C, the Martin/Hopkins equation has also been shown to be more accurate. There is a significant difference between the equations and the direct measurement method, which may lead to overtreatment or undertreatment.

## 1. Introduction

In primary and secondary coronary artery disease (CAD) prevention, low-density lipoprotein cholesterol (LDL-C) has been recognized as one of the critical variables leading to cardiovascular risk. If the goal is to make evidence-based decisions about whether a patient qualifies for LDL-C lowering therapy or if an on-treatment LDL-C level is appropriate, doctors may want to assess the accuracy of various LDL-C measurements [1,2]. As a result, precise LDL-C measurement is fundamental.

Ultracentrifugation (Beta quantification) is the gold standard for testing LDL-C. However, it is not routinely performed by clinical laboratories in western countries due to the requirement for large specimen volumes, extensive handling procedures, and long ultracentrifugation periods [3]. Instead, the Friedewald equation has typically been used to calculate LDL-C [4]. However, the Friedewald equation is less accurate in individuals with low LDL-C or high triglyceride (TG) levels, and several other methods have been developed, such as direct measurements and the Martin/Hopkins and Sampson equations. The direct approach for measuring LDL-C is advised by the Chinese guideline [5], while the 2021 practical recommendations for lipid measurement suggest using the Martin/Hopkins equation [6]. In particular, few external validations have been made using these measurements in the Asian population.

If LDL-C did not reach the target value after taking statins, ezetimibe or proprotein convertase subtilisin/kexin type 9 (PCSK9) inhibitors should be added for patients with CAD [7,8,9]. When LDL-C is accurately measured, doctors can make correct treatment decisions based on the results. Now, guidelines have pointed out that adding nonstatin therapy (i.e., PCSK9 inhibitors) is recommended for patients with ASCVD who are on maximally tolerated LDL-C lowering therapy (≥1.8 mmol/L in the American Heart Association [AHA]/American College of Cardiology [ACC] guideline; ≥1.4 mmol/L among those at very high risk in the European Society of Cardiology [ESC] guideline). Therefore, even little variations in predicted LDL-C can have a big impact on therapy choices. To explore how different methods affect treatment decisions in this high-risk population (based on American guidelines) or very high-risk populations (based on the European guidelines), we assessed the difference in estimated LDL-C among people with hypertriglyceridemia (TG ≥ 1.7mmol/L) and at various LDL-C cutpoints as mentioned above.

Using a cohort of patients with angiographically confirmed coronary artery disease, our goals in the study were to (1) quantify the discordance in LDL-C levels between equations (the Friedewald, Sampson, and Martin/Hopkins equations) and compare them with direct LDL-C measurement (dLDL-C); and (2) investigate, using these three methodologies, the percentage of patients misclassified by guidelines by direction and estimated LDL-C category.

## 2. Materials and Methods

### 2.1. Study Population

From January 2017 to December 2018, 30,349 consecutive patients who underwent coronary angiography and percutaneous coronary intervention (PCI) for CAD at Fuwai Hospital (National Center for Cardiovascular Diseases, Beijing, China) were prospectively enrolled. In this study, we retrospectively analyzed the lipid profiles, in which 51 (0.2%) patients without complete lipid panels were excluded. The study followed the Declaration of Helsinki’s standards and was approved by the hospital’s ethical review board. Before the intervention, written informed consent for prospective follow-up was obtained from all participants. Variable definitions are in Supplemental Methods.

### 2.2. Lipid Measurement and LDL-C Estimation

An automatic biochemistry analyzer determined lipid profiles in fasting status (Hitachi 7150, Tokyo, Japan). Enzymatic methods analyzed total cholesterol (TC) and TG. High-density lipoprotein cholesterol (HDL-C) concentration was measured by a homogeneous method (Determiner L HDL; Kyowa Medex, Tokyo, Japan). Utilizing the selective solubilization method, direct LDL-C (dLDL-C) was assessed (LDL-C test kit; Kyowa Medex, Tokyo, Japan). LDL-C was also estimated for all patients using three equations. The Friedewald equation:$\;LDL-C=TC\;-\;HDL-C\;-\;\left(\frac{TG}{5}\right)$. The Sampson equation [10]: $LDL-C=\frac{TC}{0.948}-\frac{HDL-C}{0.971}-\left[\frac{TG}{8.56}+\frac{TG\;\times \;non-HDL-C}{2140}–\;\left(\frac{TG^{2}}{16100}\right)-\;9.44\right].$ The Martin/Hopkins equation [11] replaces the fixed factor ‘5’ used to estimate very-low-density lipoprotein cholesterol (VLDL-C) in the Friedewald formula with one of the 180 factors (3.1–9.5) according to non-HDL-C and TG.

### 2.3. Statistical Analysis

Frequency and percentage descriptions were used to describe categorical variables. Median and IQR (25th–75th percentiles) were used to describe continuous variables. The distribution of LDL-C values per different measurements was shown in histograms. We plotted the correlation between estimated LDL-C and direct LDL-C using heated scatterplots. Concordance was defined as if the LDL-C was <1.8 mmol/L with each pairwise comparison of LDL-C equations. Estimated LDL-C that fell into the same category as dLDL-C at the following levels: <1.4, 1.4 to 1.7, 1.8 to 2.5, 2.6 to 2.9, and ≥3.0 mmol/L was considered to have been correctly categorized. The Bland–Altman approach, which plots the difference between both pressures (Y-axis) over their mean (X-axis) and displays the 95% limits of the agreement (mean difference 1.96 SD), was used to assess the differences between LDL-C equations and direct measurement. The better the agreement, the smaller the range between these two limitations [12]. It was deemed statistically significant if the 2-sided *p* < 0.05. R 4.1.2 was used to conduct each analysis.

## 3. Results

### 3.1. Baseline Characteristics

Table 1 displays the characteristics of the 30,349 patients involved in this study, 77.0% of whom were male. A total of 11,239 (37.0%) patients had hypertriglyceridemia (TG ≥ 1.7 mmol/L). The proportion of LDL-C meeting treatment goals (LDL-C < 1.8 mmol/L) was 40.8% (Friedewald equation), 35.9% (Sampson equation), 32.5% (Martin/Hopkins equation), and 26.1% (direct measurement), respectively. Supplemental Figure S1 presents LDL-C measured directly and estimated by equations does not satisfy normal distribution. The median LDL-C was 2.25 mmol/L (direct measurement), 1.98 mmol/L (Friedewald equation), 2.11 mmol/L (Martin/Hopkins equation), and 2.07 mmol/L (Sampson equation), respectively. As shown in Supplemental Figure S2, In patients with hypertriglyceridemia (TG ≥ 1.7 mmol/L), the median LDL-C was 2.48 mmol/L (direct measurement), 2.08 mmol/L (Friedewald equation), 2.39 mmol/L (Martin/Hopkins equation), and 2.25 mmol/L (Sampson equation), respectively. All three groups of patients with discordance had greater rates of hypertension, lower levels of dLDL-C and HDL-C, and higher levels of TG (Table 1).

### 3.2. Correlation of LDL-C Values among Equations and Direct Measurement

**Figure 1:**
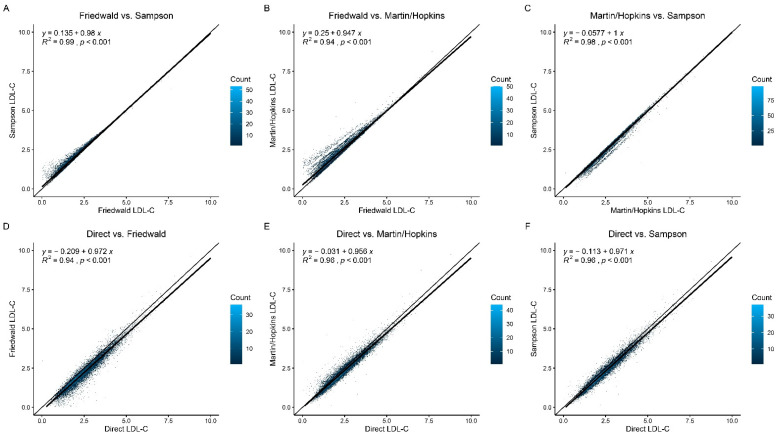
**Direct LDL-C and LDL-C equation comparison.** (**A**) Friedewald LDL-C vs. Sampson LDL-C. (**B**) Friedewald LDL-C vs. Martin/Hopkins LDL-C. (**C**) Martin/Hopkins LDL-C vs. Sampson LDL-C. (**D**) Direct LDL-C vs. Friedewald LDL-C. (**E**) Direct LDL-C vs. Martin/Hopkins LDL-C. (**F**) Direct LDL-C vs. Sampson LDL-C. LDL-C = low-density lipoprotein cholesterol.

When employing the Friedewald equation instead of the Sampson equation, the majority of patients obtained reduced LDL-C readings (Figure 1A). Similar to this, more patients using the Friedewald equation had lower LDL-C values than those with the Martin/Hopkins equation (Figure 1B). Finally, compared to the Martin/Hopkins equation, the Sampson equation lowered LDL-C in the majority of individuals (Figure 1C). Patients with hypertriglyceridemia (TG ≥ 1.7mmol/L) also showed the same patterns (Supplemental Figure S3). The Martin/Hopkins and Sampson equation demonstrated a greater connection with direct LDL-C at all levels compared to the Friedewald equation. For example, according to Friedewald, Martin/Hopkins, and the Sampson equation, the correlation coefficient between the calculated and measured LDL-C levels was 0.94, 0.96, and 0.96, respectively (Figure 1D–F).

### 3.3. The Proportion of Concordance and Discordance Using Different LDL-C Treatment Target

**Figure 2:**
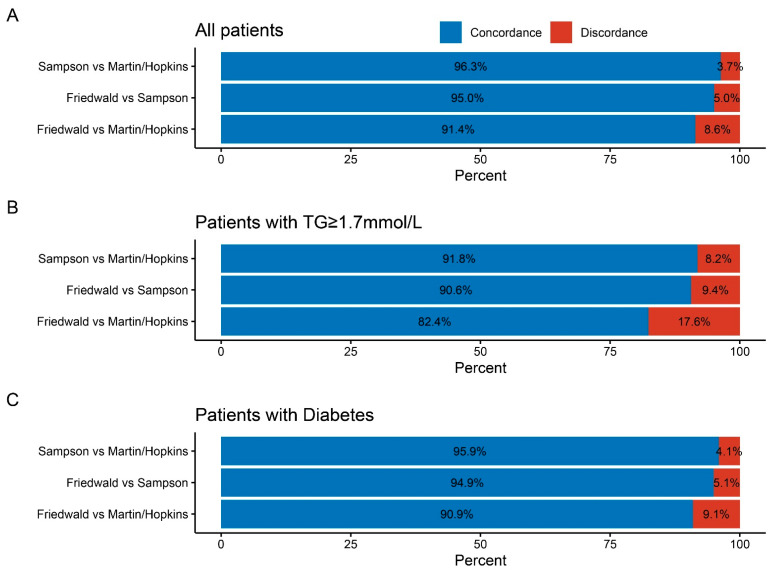
**LDL-C equation concordance and discordance at 1.8 mmol/L as the LDL-C cut point.** (**A**) Concordance and discordance in all patients. (**B**) Concordance and discordance in patients with TG ≥ 1.7mmol/L. (**C**) Concordance and discordance in patients with diabetes. LDL-C = low-density lipoprotein cholesterol.

Figure 2 shows the discordance caused by the underestimation of LDL-C levels by the Martin and Friedewald methods. Based on an LDL-C threshold of 1.8 mmol/L, the concordance is 96.3% (Sampson vs. Martin/Hopkins equation), 95.0% (Friedewald vs. Sampson), and 91.4% (Friedewald vs. Martin/Hopkins), respectively. The Sampson and Martin/Hopkins equation’s concordance is superior to that of the other combinations. The concordance proportion across the three equations (consistently estimating the LDL-C as <1.8 mmol/L) was 91.4% for all patients. However, this proportion fell to 82.4% in those with hypertriglyceridemia (TG ≥ 1.7 mmol/L). A similar trend was observed in people with diabetes.

At a lower LDL-C cutpoint of 1.4 mmol/L (Supplemental Figure S4), the proportion of concordance increased to 93.4% (Friedewald vs. Martin/Hopkins), 95.8% (Friedewald vs. Sampson), and 97.6% (Sampson vs. Martin/Hopkins), respectively. The concordance proportion increased to 86.2% in hypertriglyceridemia patients (TG ≥ 1.7 mmol/L) (Friedewald vs. Martin/Hopkins).

### 3.4. The Amount of Misclassification Used as a Direct Measurement at Various LDL-C Values

**Figure 3:**
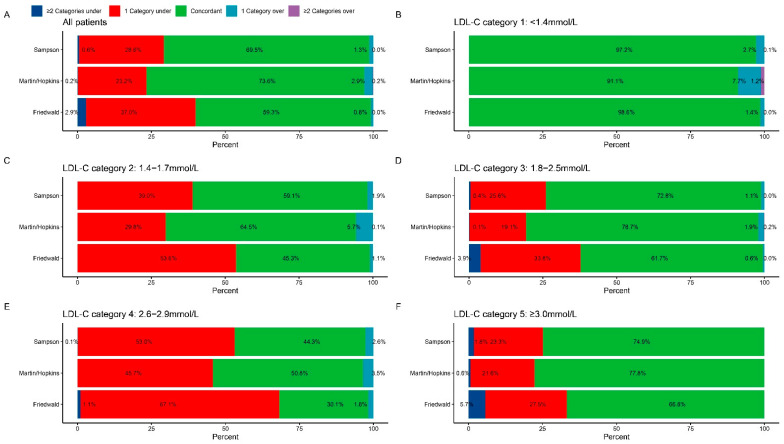
**Percentage of patients that were incorrectly classified by direction and estimated LDL-C subgroup.** (**A**) Overall. (**B**) LDL-C subgroup 1: <1.4 mmol/L. (**C**) LDL-C subgroup 2: 1.4–1.7 mmol/L. (**D**) LDL-C subgroup 3: 1.8–2.5 mmol/L. (**E**) LDL-C subgroup 4: 2.6–2.9 mmol/L. (**F**) LDL-C subgroup 5: ≥3.0 mmol/L. LDL-C = low-density lipoprotein cholesterol.

In the total cohort, 39.9% of patients who used the Friedewald equation and 29.2% of patients who used the Sampson equation in relation to direct measurement underestimated their LDL-C values when utilizing the Martin/Hopkins equation, respectively (Figure 3A). According to Friedewald, Martin/Hopkins, and Sampson, the percentage of exaggerated LDL-C classification was quite low at 0.8%, 3.1%, and 1.3%, respectively. With a proper classification rate of 73.6%, the Martin/Hopkins method outperformed the Sampson (69.5%) and the Friedewald method (59.3%) by a wide margin.

Each equation exhibits good concordance with the dLDL-C when LDL-C < 1.4 mmol/L (Figure 3B). The LDL-C classification was severely understated by the equation technique in each subgroup where LDL-C ≥ 1.4 mmol/L. When utilizing the Martin/Hopkins method, LDL-C readings between 1.4 and 1.7 mmol/L were underestimated in 29.8% of instances, compared to 39.0% when using the Sampson method and 53.6% when using the Friedewald method (Figure 3C). Compared with the direct measurement method, all three equations underestimate LDL-C partly. In contrast to the Friedewald and Sampson method, the Martin/Hopkins method had the least underclassification in each LDL-C group. Patients with diabetes and hypertriglyceridemia underwent a subgroup analysis (Supplemental Figures S5 and S6). The direct measurement approach and Martin/Hopkins equation continue to offer the best categorization consistency.

### 3.5. Agreement between Measuring and Estimating LDL-C Levels

**Figure 4:**
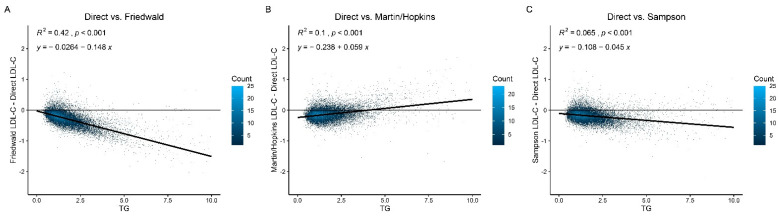
**Residual error plots for LDL-C by various equations.** (**A**) Direct LDL-C vs. Friedewald LDL-C. (**B**) Direct LDL-C vs. Martin/Hopkins LDL-C. (**C**) Direct LDL-C vs. Sampson LDL-C. LDL-C = low-density lipoprotein cholesterol. TG = triglyceride.

In our population, the Friedewald equation significantly underestimated LDL-C and had a substantially lower concordance with the dLDL-C test than the other two equations (Figure 4). The Sampson and Martin/Hopkins equations for the dLDL-C closely matched the dLDL-C data. The Martin/Hopkins and Sampson equations showed a moderate negative bias, while the Martin/Hopkins equation showed a moderate positive bias for 4.0 mmol/L or more TG levels.

The differences between the measuring and calculating LDL-C values are illustrated with Bland–Altman plots in Supplemental Figure S7. The 95% confidence limits of agreement were −0.8–0.24, −0.56–0.29, and −0.59–0.22 for the Friedewald, Martin/Hopkins, and Sampson equations, respectively. High individual variability in the Friedewald equation is reflected by a wide 95% CI. The agreement in the Martin/Hopkins and Sampson equations, on the other hand, can be regarded as ideal due to the small range of variation shown. The bias between equations and direct measurement were −0.28, −0.14, and −0.18 for the Friedewald, Martin/Hopkins, and Sampson equations, respectively. Compared with the Friedewald and Sampson equations, the Martin/Hopkins equation tended to have the minimum bias and moved only 0.1 mmol/L below the identity line.

## 4. Discussion

Our work contains various novel results to compare LDL-C levels estimated by the Friedewald, Sampson, and Martin/Hopkins methods with dLDL-C: (1) adopting a 1.8 mmol/L LDL-C cutoff, there was moderate discordance in calculated LDL-C among the three equation, (2) LDL-C calculated by three equations has more than a 20% underestimation compared to direct measurement of LDL-C, which may prevent patients from receiving intensive lipid-lowering therapy, (3) such discordance and underestimation also persisted in patients with diabetes, which was more significant in patients with hypertriglyceridemia (TG ≥ 1.7 mmol/L), (4) the LDL-C values computed using the Martin/Hopkins equation were closer to dLDL-C compared with Friedewald and Sampson LDL-C equations. The direct measurement approach and the formula method differ, and the Martin/Hopkins method is the most accurate of the three formulas. The Martin/Hopkins method could improve the identification of patients who may benefit from more intensive LDL-C lowering treatment.

### 4.1. Differences between Equations for Estimating LDL-C

With the expanding prevalence of CAD and the increasingly widespread use of PCSK9 inhibitors (e.g., Evolocumab and Alirocumab) to reduce cardiovascular risk, accurate assessment of LDL-C levels is becoming more crucial for the secondary prevention of cardiovascular disease [13,14].

In the past, the clinical standard for measuring LDL-C was the Friedewald estimation, which was produced in 1972 from 448 individuals [4]. In patients with moderate to high LDL-C levels and well-controlled TG levels (TG < 1.7mmol/L), the Friedewald estimate usually yields an appropriate result. On the other hand, it tends to significantly underestimate LDL-C values in patients with hypertriglyceridemia and low LDL-C, a population mainly beyond the Friedewald derivation sample [11,15]. In 2013, the Martin-Hopkins equation replaces the Friedewald equation’s static triglyceride denominator of 5 with a unique factor dependent on TG and non-HDL-C levels and had better accuracy [16,17]. Multiple national and international datasets have been utilized to validate the approach of Martin [18,19,20,21], including those who received PCSK9i [22,23]. The 2018 AHA/ACC blood cholesterol guideline [2], 2021 scientific statement from the National Lipid Association [6], and 2018 joint consensus panel of the European Atherosclerosis Society [24] all recommended the Martin/Hopkins equation. Sampson (2020) proposed a novel equation that estimates VLDL-C using a multiple least squares regression technique. In comparison to the Friedewald equation, the equation is more accurate for low LDL-C and TG levels between 4.5 and 9 mmol/L [10,25,26,27,28]. Regardless of the formula, LDL-C should be carefully assessed directly in TG ≥ 4.5mmol/L samples [6].

### 4.2. The Dispute over Direct Measurement

Chemical-based direct assays that do not require ultracentrifugation have evolved. Direct measures’ accuracy is debatable; various investigations have produced conflicting results [14,18,29]. 3/8 test kits satisfied the requirements of the National Cholesterol Education Program (NCEP) in healthy individuals, according to Miller’s comparison of eight direct measurements of LDL-C with ultracentrifugation. None of the eight techniques, however, were effective for patients with hyperlipidemia and atherosclerotic problems [30]. According to the contrary findings of the Miida et al. study, 4 methods including Kyowa Medex could meet NCEP standards in both primary prevention and secondary prevention population, and their performance in non-fasting status was comparable to that of fasting status [31].

### 4.3. Lipid Abnormalities in Diabetes

The most encountered lipid abnormalities in type 2 diabetes are atherogenic dyslipidemia, including increased TG, apolipoprotein B, non-HDL-C, and decreased HDL-C [32,33]. In diabetic individuals, LDL-C is also the main aim of lipid-lowering medication. Diabetic individuals are not eligible for the Friedewald techniques [34]. Our study confirmed that Martin/Hopkins method performs as well in the diabetic population as in the general population.

### 4.4. Implications for Clinical Treatment

Our results are similar to those of Sajia et al. [35]. They discovered that, in comparison to the Friedewald and Sampson equations, the Martin/Hopkins equation consistently predicted higher LDL-C values. The Friedewald vs. Martin/Hopkins comparison had a 15% discordance rate, the Friedewald vs. Sampson comparison had a 9% discordance rate, and the Sampson vs. Martin/Hopkins comparison had a 7% discordance rate. When the Friedewald, Martin/Hopkins, and Sampson methods were evaluated in our large clinical dataset, we also found the discordance between equations and direct LDL-C measurement in the LDL-C category. The Martin/Hopkins equation is considerably closer to a direct measurement. However, the differences between the three estimated LDL-C formulas were much more minor in Asian populations than in the white populations.

Significant therapeutic ramifications result from the underestimate of LDL-C by the Friedewald and Sampson equations at low LDL-C cutpoints. According to the findings of our investigation, using the Friedewald and Sampson methods in CAD and hypertriglyceridemia patients is likely to underestimate LDL-C significantly. This implicates that an LDL-C predicted by the Friedewald formula meets the target when it does not actually, which is a level that should receive more intensive LDL-C lowering therapy. This suggests that the Friedewald’s estimated LDL-C prediction was lower than the dLDL-C value, undertreating people at higher risk of ischemic events. In addition, these situations are more common in those at a higher risk of cardiovascular disease (those with obesity, diabetes, hypertriglyceridemia, or metabolic syndrome) [20]. On the other hand, the Martin/Hopkins equation might help identify more individuals who could benefit from LDL-C lowering treatment that is more intensive.

More importantly, in our population, the underestimation of calculated LDL-C by all three methods exceeds 20%. Using the equations as a criterion, 20% of patients may miss out on intensive lipid-lowering therapy. If we use direct measures as the standard, 20% of patients may have received overtreatment. Given the potential cardiovascular benefits and the very low LDL-short-term C’s safety profile [36], achieving lower levels in certain high-risk patients may be helpful. There are significant differences in LDL-C measurement methods between China and the Occident. Without agreement on LDL-C measurement methods, it is impossible to discuss whether LDL-C can reach the standard.

Due to the slightly varied physical definitions of lipoprotein particles used by each of the aforementioned “LDL-cholesterol” estimation techniques, the results obtained from the same sample can vary [37]. At present, LDL-C is still the primary target of lipid-lowering therapy. Due to their reasonable accuracy and minimal likelihood of underestimating LDL-C, we believe the Martin/Hopkins approach to be the most useful for determining LDL-C. We also need to confirm the accuracy of direct LDL-C measurement.

### 4.5. Study Limitations

First, we did not compare trueness to the LDL-standard C’s quantification measurement method. Second, LDL-C was measured only once at admission. However, we used a large sample size of CAD patients undergoing PCI from a single-center [38,39] and sought out routine medical attention was representative of daily routine practice in the Asian community in the real world. In further research, we will need to externally validate our results across a broad range of TG levels. There is also a need to look into the implications of different LDL-C measures on cholesterol-lowering medication and consequent changes in CAD prognosis.

## 5. Conclusions

Using a 1.8 mmol/L LDL-C cutoff and comparing it to the validated Martin/Hopkins equation, the Friedewald equation produced the lowest levels of LDL-C, followed by the Sampson equation. Those with hypertriglyceridemia (TG ≥ 1.7mmol/L) had a higher proportion of discordance in estimated LDL-C. In the classification of LDL-C, the Martin/Hopkins equation was also shown to be more accurate in CAD patients undergoing PCI. There is a significant difference between the equations and the direct measurement method, which may lead to overtreatment or undertreatment. Validating the performance of direct measurements in large populations may be valuable and relevant to the guidelines.

## Figures and Tables

**Table 1:** Differences in patient characteristics between concordant and discordant groups.

	**All**	**Friedewald vs. Sampson Equation**			**Friedewald vs. Martin/Hopkins Equation**			**Martin/Hopkins vs. Sampson Equation**		
		**Discordant ^a^**	**Concordant**	***p***	**Discordant ^b^**	**Concordant**	***p***	**Discordant ^c^**	**Concordant**	***p***
n	30349	1512	28837		2612	27737		1125	29224	
Age, y	59.76 (10.21)	58.45 (10.34)	59.83 (10.20)	<0.001	57.88 (10.36)	59.94 (10.18)	<0.001	57.29 (10.36)	59.86 (10.19)	<0.001
Male	23370 (77.0)	1165 (77.1)	22205 (77.0)	0.99	2057 (78.8)	21313 (76.8)	0.028	911 (81.0)	22459 (76.9)	0.001
Hypertension	20533 (67.7)	1062 (70.2)	19471 (67.5)	0.03	1841 (70.5)	18692 (67.4)	0.001	795 (70.7)	19738 (67.5)	0.03
Diabetes	13903 (45.8)	706 (46.7)	13197 (45.8)	0.496	1262 (48.3)	12641 (45.6)	0.003	565 (50.2)	13338 (45.6)	0.003
Tobacco use	17858 (58.8)	912 (60.3)	16946 (58.8)	0.242	1607 (61.5)	16251 (58.6)	0.004	708 (62.9)	17150 (58.7)	0.005
HF	4119 (13.6)	199 (13.2)	3920 (13.6)	0.66	328 (12.6)	3791 (13.7)	0.12	131 (11.6)	3988 (13.6)	0.06
CKD	1940 (6.4)	119 (7.9)	1821 (6.3)	0.018	188 (7.2)	1752 (6.3)	0.086	72 (6.4)	1868 (6.4)	1
FH	42 (0.1)	0 (0.0)	42 (0.1)	0.258	0 (0.0)	42 (0.2)	0.086	0 (0.0)	42 (0.1)	0.388
CAD										
Current/prior MI	10509 (34.6)	518 (34.3)	9991 (34.6)	0.779	904 (34.6)	9605 (34.6)	1	394 (35.0)	10115 (34.6)	0.801
CVD	4672 (15.4)	225 (14.9)	4447 (15.4)	0.596	372 (14.2)	4300 (15.5)	0.093	155 (13.8)	4517 (15.5)	0.137
PAD	3678 (12.1)	179 (11.8)	3499 (12.1)	0.762	294 (11.3)	3384 (12.2)	0.167	119 (10.6)	3559 (12.2)	0.117
Lipid values, mmol/L										
TC	3.86 [3.27, 4.60]	3.76 [3.53, 4.10]	3.87 [3.25, 4.63]	0.064	3.71 [3.47, 4.02]	3.90 [3.24, 4.66]	<0.001	3.63 [3.41, 3.93]	3.88 [3.26, 4.63]	<0.001
TG	1.45 [1.08, 2.01]	2.10 [1.59, 2.95]	1.42 [1.06, 1.96]	<0.001	2.25 [1.71, 3.26]	1.40 [1.05, 1.91]	<0.001	2.42 [1.85, 3.53]	1.42 [1.07, 1.96]	<0.001
HDL-C	1.07 [0.90, 1.27]	1.00 [0.86, 1.16]	1.07 [0.91, 1.27]	<0.001	0.96 [0.83, 1.12]	1.08 [0.91, 1.28]	<0.001	0.92 [0.80, 1.07]	1.08 [0.91, 1.28]	<0.001
Non–HDL-C	2.74 [2.19, 3.46]	2.68 [2.48, 3.03]	2.75 [2.17, 3.48]	0.018	2.67 [2.48, 3.00]	2.76 [2.15, 3.51]	0.041	2.63 [2.47, 2.96]	2.75 [2.17, 3.48]	0.829
Friedewald LDL-C	1.98 [1.51, 2.60]	1.73 [1.66, 1.77]	2.03 [1.49, 2.64]	<0.001	1.67 [1.52, 1.75]	2.07 [1.51, 2.67]	<0.001	1.53 [1.33, 1.64]	2.02 [1.53, 2.63]	<0.001
Sampson LDL-C	2.07 [1.60, 2.70]	1.86 [1.83, 1.92]	2.11 [1.58, 2.74]	<0.001	1.81 [1.75, 1.88]	2.15 [1.57, 2.77]	<0.001	1.73 [1.66, 1.77]	2.10 [1.59, 2.73]	<0.001
Martin/Hopkins LDL-C	2.11 [1.66, 2.74]	1.95 [1.90, 2.13]	2.14 [1.63, 2.78]	<0.001	1.92 [1.86, 2.04]	2.18 [1.62, 2.80]	<0.001	1.86 [1.83, 1.94]	2.15 [1.64, 2.77]	<0.001
Direct LDL-C	2.25 [1.77, 2.89]	2.09 [2.00, 2.22]	2.28 [1.75, 2.93]	<0.001	2.02 [1.90, 2.15]	2.32 [1.74, 2.97]	<0.001	1.93 [1.82, 2.03]	2.29 [1.77, 2.92]	<0.001

Values are mean ± SD, n (%), or median (IQR). Abbreviations as in Table 1. ^a^ Discordant defined as Friedewald LDL-C of <1.8 mmol/L and Sampson equation LDL-C of ≥1.8 mmol/L. ^b^ Discordant defined as Friedewald LDL-C of <1.8 mmol/L and Martin/Hopkins equation LDL-C of ≥1.8 mmol/L. ^c^ Discordant defined as Sampson equation LDL-C of <1.8 mmol/L and Martin/Hopkins equation LDL-C of ≥1.8 mmol/L.

## Data Availability

Our datasets are available from the corresponding author upon reasonable request after approval from the Institutional Review Board of State Key Laboratory of Cardiovascular Disease, Fuwai Hospital, National Center for Cardiovascular Diseases due to ethical limitations related to the consent provided by subjects at the time of study commencement.

## Supplementary Materials

The following supporting information can be downloaded at: https://www.mdpi.com/article/10.3390/jcdd9100342/s1, Supplemental Figure S1: Distribution of LDL-C values per measurements; Supplemental Figure S2: Distribution of LDL-C values per measurements in patients with TG levels of >= 1.7 mmo/L; Supplemental Figure S3: Comparison of direct LDL-C and LDL-C equations with TG Levels of >= 1.7 mmol/L; Supplemental Figure S4: Concordance and discordance between LDL-C equations at an LDL-C cut point of 1.4 mmol/L; Supplemental Figure S5: Proportion of misclassified patients per direction by estimated LDL-C category in patient with hypertriglyceridemia; Supplemental Figure S6: Proportion of misclassified patients per direction by estimated LDL-C category in patient with diabetes; Supplemental Figure S7: Bland-Altman plots of the bias between estimated LDL-C and direct LDL-C.

## Abbreviations

CAD	coronary artery disease
LDL-C	low-density lipoprotein cholesterol concentration
TG	triglycerides
PCSK9	proprotein convertase subtilisin/kexin type 9
AHA	American Heart Association
ACC	American College of Cardiology
ESC	European Society of Cardiology
TC	total cholesterol
dLDL-C	direct LDL-C
PCI	percutaneous coronary intervention
eGFR	estimated glomerular filtration rate
FH	familial hypercholesterolemia
DLCNS	Dutch Lipid Clinic Network Score
PAD	peripheral artery disease
HDL-C	high-density lipoprotein cholesterol
VLDL-C	very-low-density lipoprotein cholesterol
IQR	interquartile range
NCEP	National Cholesterol Education Program
