# Supplementary material for: Comparison of Estimated LDL Cholesterol Equations with Direct Measurement in Patients with Angiographically Confirmed Coronary Artery Disease
Source: J Cardiovasc Dev Dis. 2022 Oct 7;9(10):342. doi: 10.3390/jcdd9100342 (PMC9604394; doi:10.3390/jcdd9100342)
Supplement: Supplementary file 1 [file jcdd-09-00342-s001.zip › jcdd-1923768-supplementary.pdf]

Supplemental methods

Variables' definitions

Hypertension was defined as self-reported hypertension, currently taking antihypertensive drugs, or recorded systolic blood pressure >= 140 mmHg or diastolic blood pressure >= 90 mmHg three or more consecutive times. Diabetes was diagnosed by fasting plasma glucose >= 7.0 mmol/L, the two-hour plasma glucose of the oral glucose tolerance test >= 11.1 mmol/L, those with hemoglobin A1c (HbA1c) >= 6.5% at baseline, or current use of hypoglycemic drugs or insulin. The diagnostic algorithm for heart failure is consistent with the 2021 ESC Guidelines for the diagnosis and treatment of acute and chronic heart failure[9]. The estimated glomerular filtration rate (eGFR) was calculated according to the MDRD GFR equation, and patients with eGFR of <60 ml/min/1.73m^2 were diagnosed with CKD. Familial hypercholesterolemia (FH) was diagnosed by Dutch Lipid Clinic Network Score (DLCNS) for FH. Cerebrovascular disease includes stroke, carotid stenosis, vertebral stenosis, intracranial stenosis, aneurysms, and vascular malformations. Peripheral artery disease (PAD) was defined as a history of surgical or percutaneous peripheral artery revascularization or a stenosis >= 50% at Doppler ultrasound imaging in a peripheral artery district (extracranial carotids or lower limbs).

Supplemental figures

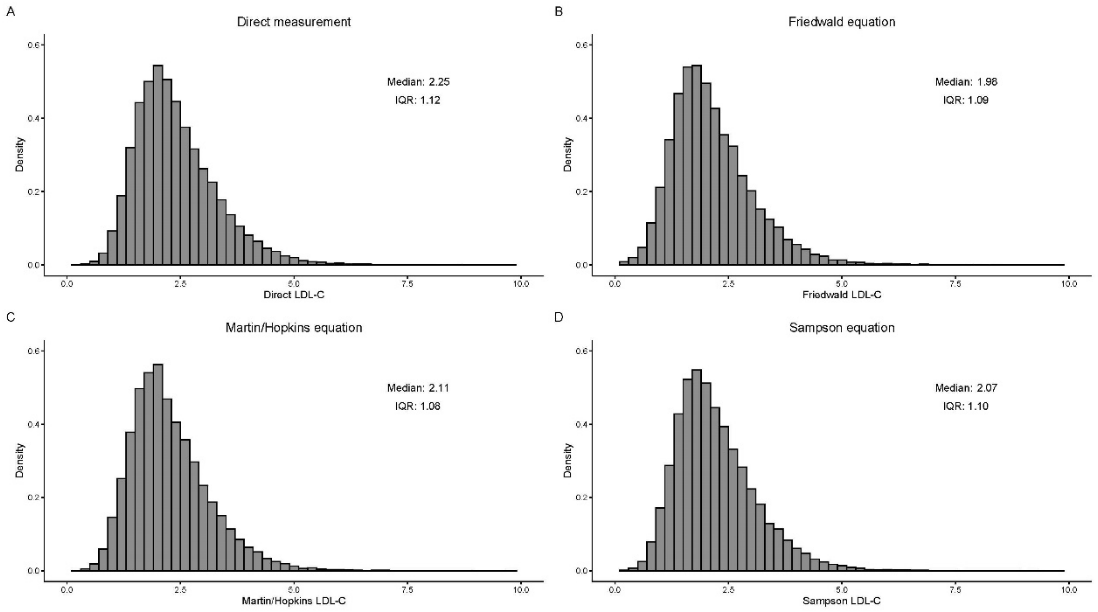

Supplemental Figure S1. Distribution of LDL-C values per measurements.
Distribution of LDL-C values (mmol/L) estimated by (A) direct measurement, (B) the Friedewald, (C) Martin/Hopkins, and (D) Sampson equations. LDL-C = low-density lipoprotein cholesterol.

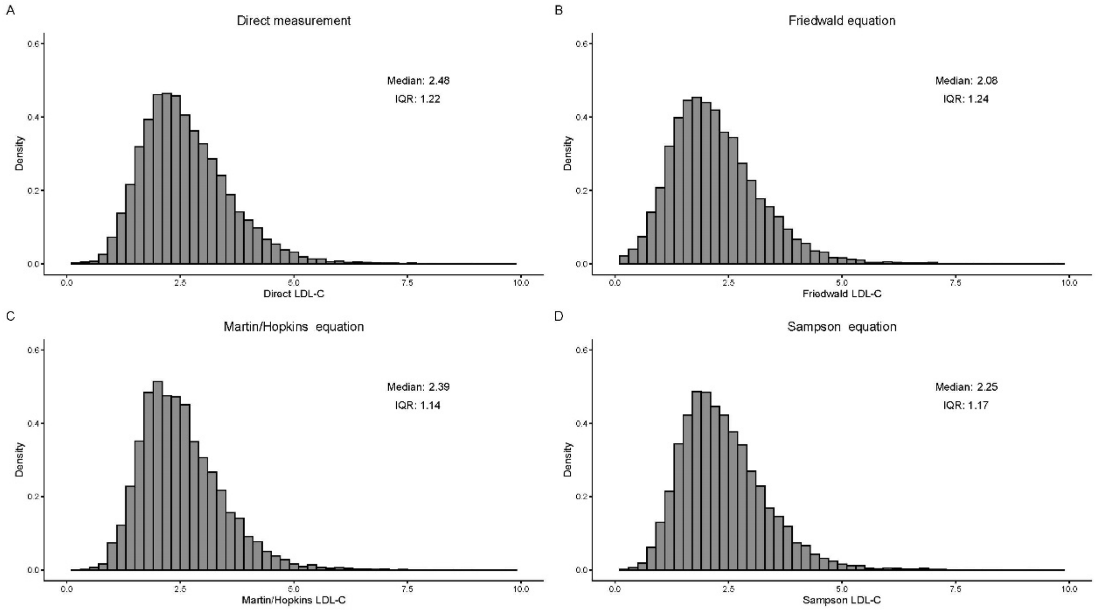

Supplemental Figure S2. Distribution of LDL-C values per measurements in patients with TG levels of >= 1.7 mmo/L.

Distribution of LDL-C values (mmol/L) estimated by (A) direct measurement, (B) the Friedewald, (C) Martin/Hopkins, and (D) Sampson equations. LDL-C = low-density lipoprotein cholesterol.

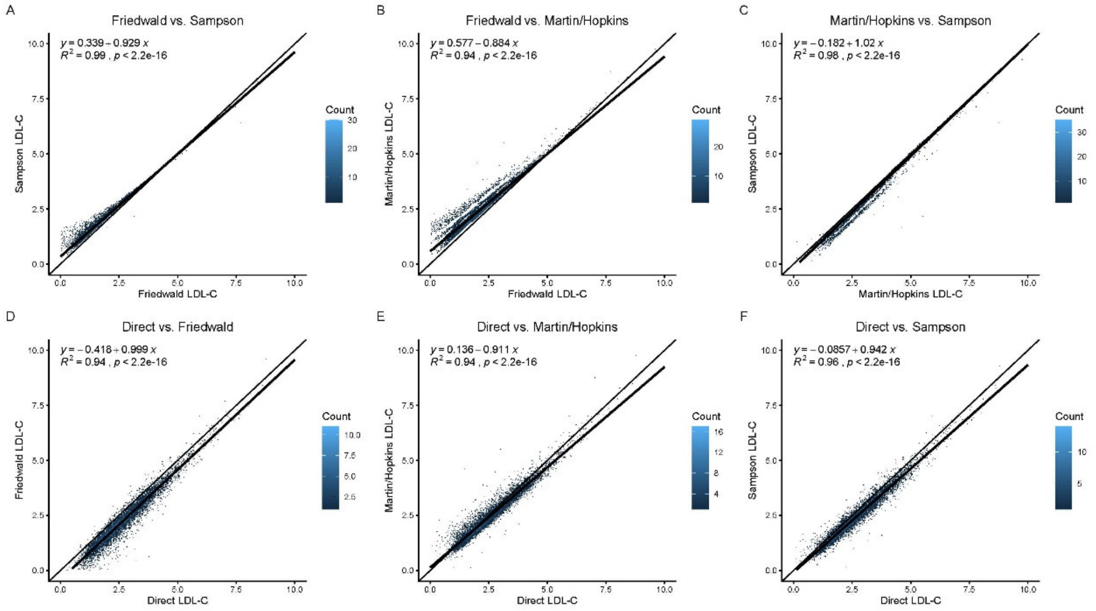

Supplemental Figure S3. Comparison of direct LDL-C and LDL-C equations with TG Levels of >= 1.7 mmol/L.

(A) Friedewald vs. Sampson equation. (B) Friedewald vs. Martin/Hopkins equation. (C) Martin/Hopkins vs. Sampson equation. (D) Direct measurement vs. Friedewald equation. (E) Direct measurement vs. Martin/Hopkins equation. (F) Direct measurement vs. Sampson equation. The diagonal line is the unity line in each graph, where both equations estimate the same value. Each dot represents the estimated LDL-C by the respective equation indicated on the x- and y-axes. The dot's color represents data density from light blue to dark blue. LDL-C = low-density lipoprotein cholesterol.

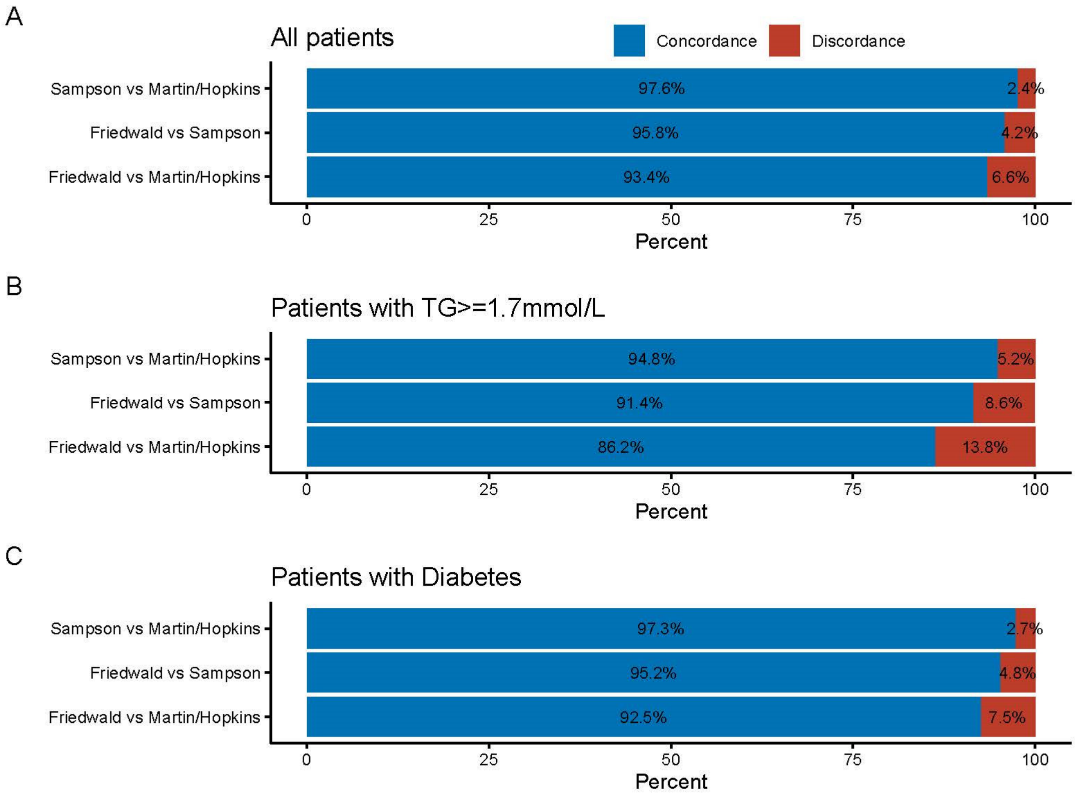

Supplemental Figure S4. Concordance and discordance between LDL-C equations at an LDL-C cut point of 1.4 mmol/L.

Percentages of patients with concordant and discordant LDL-C values estimated by the 3 equations. (A) Concordance and discordance in all patients. (B) Concordance and discordance in patients with TG>= 1.7mmol/L. (C) Concordance and discordance in patients with diabetes. LDL-C = low-density lipoprotein cholesterol.

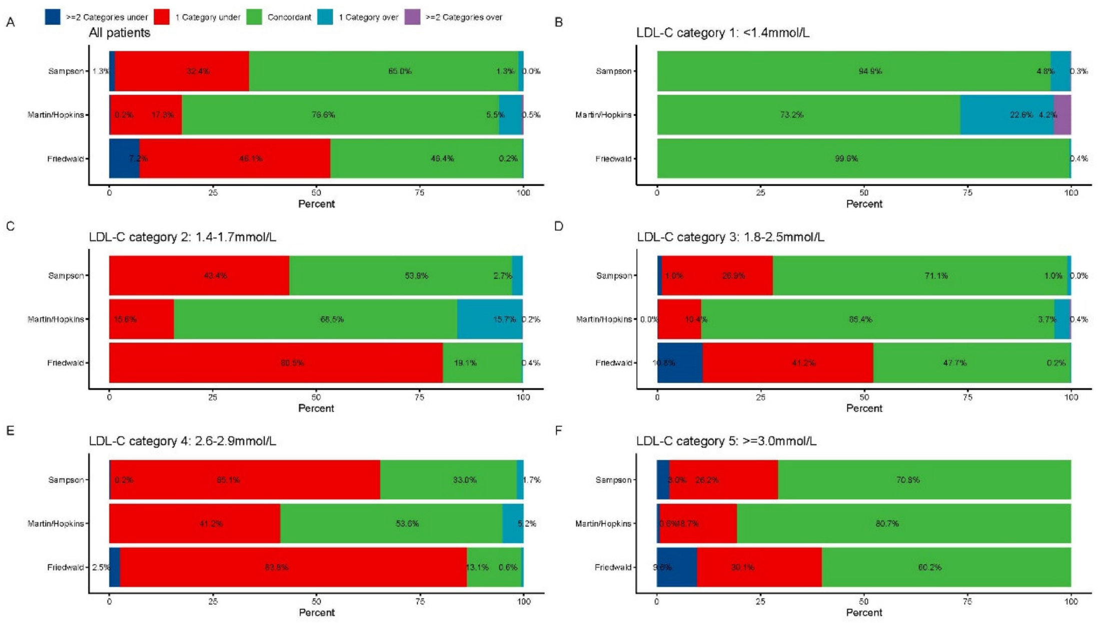

Supplemental Figure S5. Proportion of misclassified patients per direction by estimated LDL-C category in patient with hypertriglyceridemia.

Graphs represent the total percentage of under classified and overclassified patients within each LDL-C category. Values to the left and right of 0 on the x-axis indicate percentage under classified and percentage overclassified, respectively. Proportion of misclassification in all patients (A), LDL-C category 1 :<1.4 mmol/L (B), LDL-C category 2 :1.4-1.7 mmol/L (C), LDL-C category 3 :1.8-2.5 mmol/L (D), LDL-C category 4 :2.6-2.9 mmol/L (E), and LDL-C category 5 :>=3.0 mmol/L (F). LDL-C = low-density lipoprotein cholesterol.

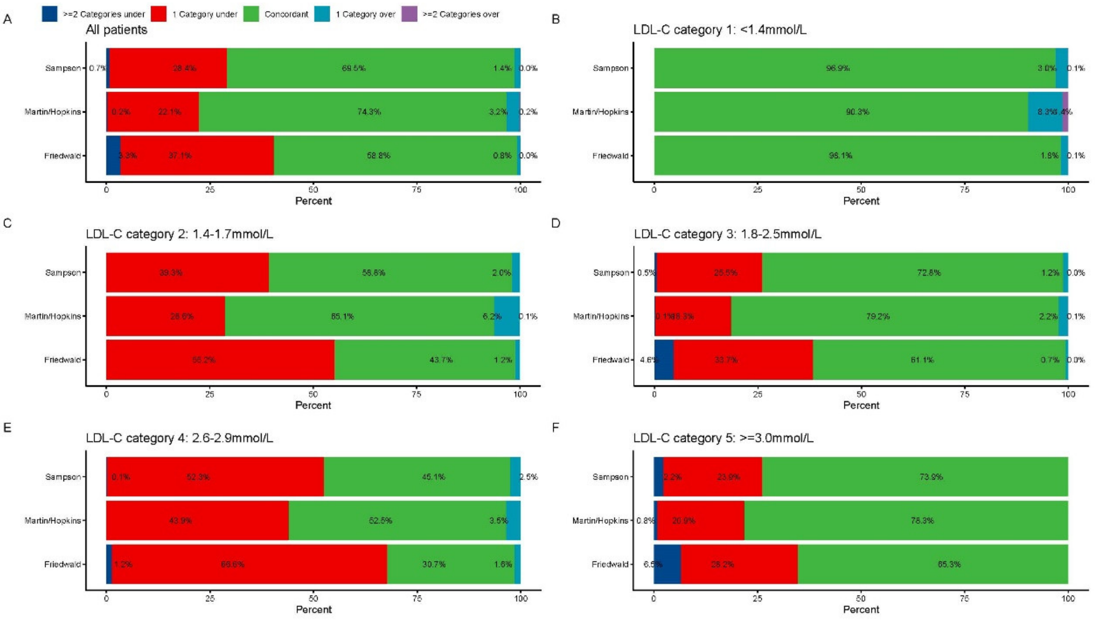

Supplemental Figure S6. Proportion of misclassified patients per direction by estimated LDL-C category in patient with diabetes.

Graphs represent the total percentage of under classified and overclassified patients within each LDL-C category. Values to the left and right of 0 on the x-axis indicate percentage under classified and percentage overclassified, respectively. Proportion of misclassification in all patients (A), LDL-C category 1 :<1.4 mmol/L (B), LDL-C category 2 :1.4-1.7 mmol/L (C), LDL-C category 3 :1.8-2.5 mmol/L (D), LDL-C category 4 :2.6-2.9 mmol/L (E), and LDL-C category 5 :>=3.0 mmol/L (F). LDL-C = low-density lipoprotein cholesterol.

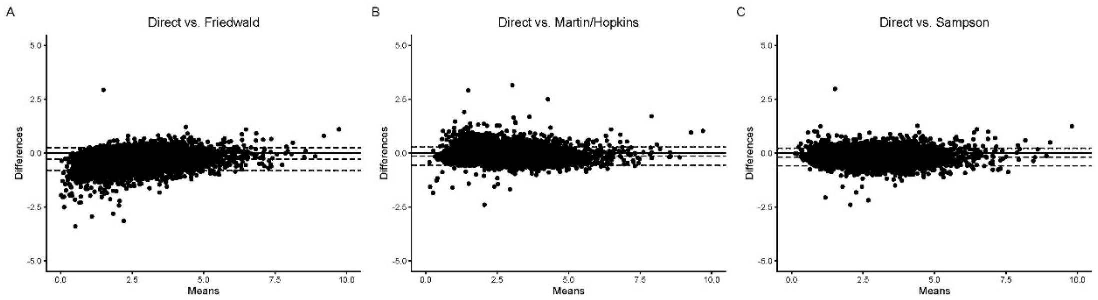

Supplemental Figure S7. Bland-Altman plots of the bias between estimated LDL-C and direct LDL-C.

(A) Direct measurement vs. Friedewald equation. (B) Direct measurement vs. Martin/Hopkins equation. (C) Direct measurement vs. Sampson equation. LDL-C = low-density lipoprotein cholesterol.
